# HIV-1 RNA Dysregulates the Natural TLR Response to Subclinical Endotoxemia in Kenyan Female Sex-Workers

**DOI:** 10.1371/journal.pone.0005644

**Published:** 2009-05-21

**Authors:** Richard T. Lester, Xiao-Dan Yao, T. Blake Ball, Lyle R. McKinnon, Were R. Omange, Rupert Kaul, Charles Wachihi, Walter Jaoko, Kenneth L. Rosenthal, Francis A. Plummer

**Affiliations:** 1 Department of Medical Microbiology and Infectious Diseases, University of Manitoba, Winnipeg, Manitoba, Canada; 2 Department of Medical Microbiology, University of Nairobi, Nairobi, Kenya; 3 Department of Pathology & Molecular Medicine, McMaster University, Hamilton, Ontario, Canada; 4 National Microbiology Laboratory, Public Health Agency of Canada, Winnipeg, Manitoba, Canada; 5 Department of Medicine, University Health Network and University of Toronto, Toronto, Ontario, Canada; New York University School of Medicine, United States of America

## Abstract

**Background:**

Subclinical endotoxemia has been reported in HIV-1 infected persons and may drive systemic immune activation and pathogenesis. Proinflammatory responsiveness to endotoxin (LPS) is mediated by Toll-like receptor 4 (TLR4). We therefore examined the association between plasma LPS levels, HIV RNA, and TLR4 expression and cytokine responses in the blood of HIV infected and uninfected participants in a cohort of female sex-workers in Kenya.

**Methodology/Principal Findings:**

Ex vivo plasma and peripheral blood mononuclear cells (PBMC) were assessed for LPS and TLR mRNA, respectively. The effects of HIV single stranded RNA, a TLR8 ligand, on TLR4 and LPS signaling were further assessed in short term PBMC culture. Both HIV uninfected and infected subjects frequently had low detectable LPS levels in their plasmas. Significantly increased LPS levels were associated with chronic HIV-1 infection, both treated and untreated, but not with other acute or semi-chronic conditions reported. In HIV-uninfected subjects, TLR4 mRNA expression levels correlated inversely with plasma LPS levels, suggesting chronic endotoxin ‘tolerance’ in vivo. A similar effect of reduced TLR4 mRNA was seen in short term PBMC culture after stimulation with LPS. Interestingly, the apparent in vivo tolerance effect was diminished in subjects with HIV infection. Additionally, pre-stimulation of PBMC with LPS lead to proinflammatory (TNF-α) tolerance to subsequent LPS stimulation; however, pre-treatment of PBMC with HIV single-stranded RNA40, could enhance TLR4-mediated LPS responsiveness in vitro.

**Conclusions/Significance:**

Thus, dysregulation of endotoxin tolerance by HIV-1 RNA may exacerbate HIV chronic immune activation and pathogenesis.

## Introduction

Lipopolysaccharide (LPS or endotoxin) is a component of Gram negative bacterial cell walls and is known to induce large proinflammatory responses that cause bacterial sepsis[1]. Recently, increased levels of LPS have been found in the plasma of chronically HIV-1 infected individuals who did not demonstrate overt signs of sepsis [2]. The source of the circulating endotoxin is thought to be from ‘microbial translocation’ of bacterial products across the gastrointestinal tract since HIV and SIV are known to induce dramatic cellular changes in the gastrointestinal mucosa [3], [4]. This has not, however, been explored in African populations where enteric microflora and opportunistic infections may be different and the HIV pandemic is at its peak. Whatever the endotoxin source, subclinical endotoxemia could be a substantial driving force for chronic immune activation in HIV which has been linked to disease progression[5].

Microbes that invade the external physical barriers of mammalian hosts are recognized instantly by sensors of the innate immune system. Toll-like receptors (TLR), for instance, which are embedded in host immune cell membranes and intracellular compartments, recognize these microbial or pathogen associated molecular patterns (PAMPs)[6]. LPS was the prototypic proinflammatory microbial signaling molecule shown to induce the mammalian *Toll* pathway[7], and the receptor was later identified as TLR4. More recently, short GU rich sequences of HIV-1 RNA were the first described natural ligands for TLR7 and TLR8[8], [9], [10]. Many human cell types express various TLR, especially those that have innate immune or barrier function. Peripheral blood mononuclear cells (PBMC) are composed of circulating immune cells including lymphocytes, monocytes, and dendritic cells that are rich in TLR expression[11] and respond broadly to TLR ligands. Both LPS and HIV ssRNA have been linked to elevated immune activation levels in chronic HIV infection[2], [12].

We recently reported that mRNA expression of multiple TLRs, including TLR4, TLR7 and TLR8, are significantly increased in PBMCs of subjects chronically infected with HIV-1[13]. A proinflammatory consequence is inferred, since TNF-α responses induced by LPS and HIV ssRNA were also higher in PBMC isolated from HIV infected subjects than from uninfected controls. We also identified a weak association between the increased TLR expression and plasma viral load, suggesting that viral products or viral replication may drive these TLR signaling changes. We hypothesized that HIV ssRNA may play a role in regulating TLR signaling. In this study, we sought to confirm the association between HIV infection and sub-clinical endotoxemia in a cohort of female sex-workers in Kenya, and evaluate TLR-mediated signaling. We measured plasma endotoxin levels matched to TLR expression levels and conducted in vitro studies to further evaluate the effect of LPS and ssRNA on cross TLR proinflammatory signaling.

## Materials and Methods

### Human subjects

HIV-1 infected and HIV uninfected women were recruited from the Pumwani sex-worker cohort in Nairobi, Kenya [14]. All subjects gave informed written consent and were sampled during scheduled outpatient research visits. HIV infected subjects tested seropositive on at least two HIV ELISA tests, at least 6 months apart. Routine questionnaires including data on clinical symptomatology, physician exam, and basic laboratory results were collected on each visit. The study was approved by the Institutional Review Boards of The University of Manitoba, and the University of Nairobi through the Kenyatta National Hospital ethical review committee. All clinical investigation was conducted according to the principles of the Helsinki Declaration.

### Sample acquisition and preparation

Peripheral blood was collected by venipuncture. Routine CD4 T cell counts were performed on all subjects using flow cytometry (FACScan, BD) and available plasma viral loads were measured by polymerase chain reaction (Roche bDNA). Cryopreserved plasmas, matched to our previous TLR study[13], were used in endotoxin assays. For new samples, peripheral blood mononuclear cells (PBMCs) were isolated from fresh blood by density gradient centrifugation as previously described[13] and were used fresh for *in vitro* culture studies or aliquotted and frozen in Trizol for TLR quantitative reverse-transcriptase real time PCR (QRT-PCR).

### Endotoxin assay

Plasma was first centrifuged to remove debris then diluted to 20% with endotoxin-free water and heated to 70°C for 10 min to inactivate plasma proteins as previously described[2]. Plasma LPS levels were then quantified using a commercially available Limulus Amebocyte assay (Associates of Cape Cod) and analyzed with background subtracted according to the package insert.

### TLR expression

Total RNA was extracted from TRIzol samples containing approximately 1×10^6^ PBMCs as per manufacturer instructions (Invitrogen). The detailed protocol and TaqMan primers are published elsewhere[13]. Readout of data was a ratio of the gene quantity to its 18S rRNA quantity, defined as relative expression.

### In vitro TLR stimulations

Freshly isolated PBMCs were cultured at 1×10^6^ cell/ml in RPMI and either 0.1 ug/ml LPS (Invivogen), 1 ug/ml ssRNA40 (Invivogen), or media alone (RPMI and 10%FCS) for most studies. For TLR-ligand ‘priming’ studies, supernatants from PBMC culture were removed after 16 hours priming with either LPS or ssRNA40, or media control. The primed cells were then washed twice in RPMI, and replenished with fresh media and cultured with LPS for a further 8 hours (post-prime LPS stimulation). Additional priming studies were carried out using lower dose of LPS (0.01 ug/ml) to examine a dose effect. Post-prime media control assays ruled out significant cytokine carry-over from initial TLR-ligand stimulations. Culture supernatants were diluted 10-fold and assayed for cytokines using cytometric bead array (BD Biosystems) on a FACScan flow cytometer (BD) as per the manufacturers protocol. Cell pellets for Q-PCR TLR expression studies were immediately frozen in Trizol.

### Statistical analysis

Data were analyzed using SPSS, Microsoft Excel and Prism 5.0 software (GraphPad). Data from the FAScan (BD) was acquired using the BD CBA acquisition software into Microsoft Excel and the analyzed by Prism 4.0 software. Non-parametric tests were used including Mann-Whitney tests for comparing independent variables, Wilcoxon rank *t*-tests for paired samples, unpaired t tests for replicate experiments, and Spearman's rank test for matched correlations. Chi squared (χ^2^) tests were used for analysis of categorical variables (detectable endotoxemia) and associated relative risks (RR) reported. Two tailed tests were used in all analyses.

## Results

### Increased plasma endotoxin levels are associated with HIV infection but not acute presenting conditions

Study participant characteristics for direct *ex vivo* studies are shown in Table 1. Matched plasmas to PBMC were available from HIV-uninfected (n = 31), HIV-infected ART-naïve (n = 38), and HIV-infected on antiretroviral therapy (ART) (n = 19) women. Age and the level of sex-work activity were similar among these groups. The CD4 T cell counts of the women taking ART were lower than untreated subjects (P = 0.008), due to the advanced stages of disease in subjects initiating ART and insufficient time for adequate CD4 T cell recovery (Median duration on ART 14 months, range 1–42). However, there was substantive suppression of plasma HIV viral loads (mean drop 1.49 log10 copies/ml, P<0.001) in subjects taking ART.

**Table 1:** Characteristics of subjects participating in the *in vivo* endotoxin studies, from the Pumwani sex-worker cohort.

	HIV−		HIV+		ART	
No. Subjects: n	31		38		19	
Age: mean (range)	40.1	(24–62)	37.6	(27–62)	41.4	(25–62)
Sex clients per day: mean (range)	4.4	(1–11)	5.1	(1–10)	3.9	(1–8)
CD4 count : mean (std dev.)	1,126	(318)	443	(253)	301	(131)
HIV RNA load: mean Log10VL, std dev.)	n/a	(n/a)	3.5	(.99)	2.0	(0.80)

**Figure 1:**
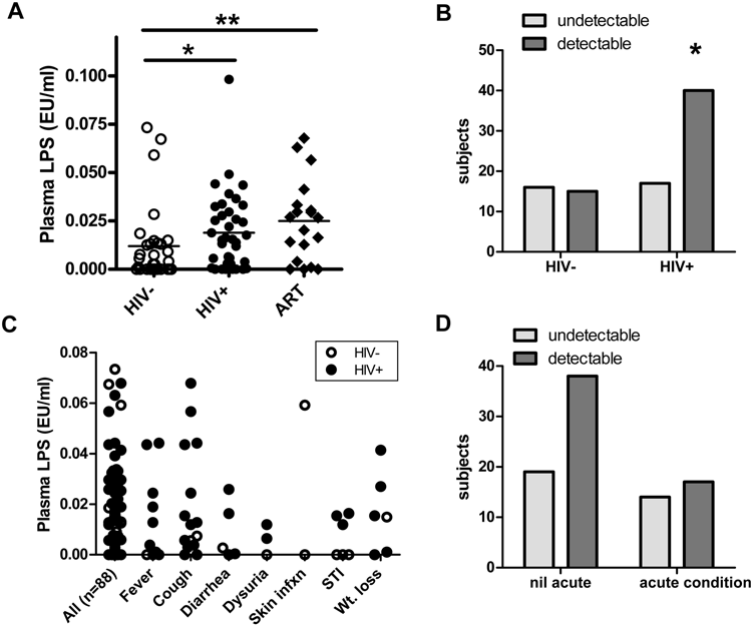
Plasma endotoxin (LPS) levels according to HIV status or presenting clinical condition. (A) Chronic HIV-1 infection was significantly associated with increased plasma LPS levels in both untreated (Mean 0.0188EU/ml, P = 0.023) and ART treated female sex-workers (Mean 0.0249, P = 0.009) compared to uninfected controls (Mean 0.0119 EU/ml). (B) HIV infected subjects were also more likely to have detectable LPS plasma levels than uninfected subjects (limit of detection 0.005 EU/ml, RR = 1.45, P = 0.048). (C) Symptomatic clinical conditions suggestive of acute infectious etiologies were more frequent in HIV infected subjects than uninfected subjects. (D) However, the acutely reported or diagnosed conditions were not independently associated with high plasma endotoxin levels (RR = 0.823, P = 0.274). Mann Whitney tests were used for comparisons between groups, and Chi-squared for Relative Risk approximations (RR). Two tailed tests were used with level of significance *P<0.05, **P<0.01.

Plasma endotoxin levels were frequently detectable by LAL in both HIV infected and uninfected subjects (level of detection 0.005 EU/ml). We confirmed that LPS levels were higher in plasma from HIV-1 infected subjects compared with HIV-1 uninfected subjects, which was true regardless of whether they were receiving ART (Figure 1A, Mean plasma endotoxin levels for HIV-negative = 0.0119±0.0196 EU/ml, HIV-positive untreated = 0.0188±0.0199 EU/ml, P = 0.022, and HIV-positive on ART = 0.0249±0.0210 EU/ml, P = 0.009)). In this study, LPS levels did not correlate with plasma viral load (R = −0.0839, P = 0.593) nor CD4 T cell counts (R = 0.0122, P = 0.929).

The proportion of subjects with detectable endotoxemia was also higher among subjects with HIV infection than those without (Figure 1B, RR = 1.45, P = 0.048). Endotoxin levels in acute symptomatic or physician diagnosed conditions during the study visits are shown in Figure 1C. Although the study was not powered to evaluate acute presenting conditions individually, there was no trend toward increased endotoxemia when the acute conditions were grouped (Figure 1D, RR = 0.823, P = 0.27). In fact, a higher proportion of HIV infected subjects with detectable endotoxemia did not have symptoms compared to those that did not have detectable endotoxemia, though this was not statistically significant. To summarize, in these women of high infection-risk, chronic HIV-1 infection, whether treated or untreated, was associated with endotoxemia that was unassociated with acute presentation of illness.

### TLR signaling and endotoxin tolerance

**Figure 2:**
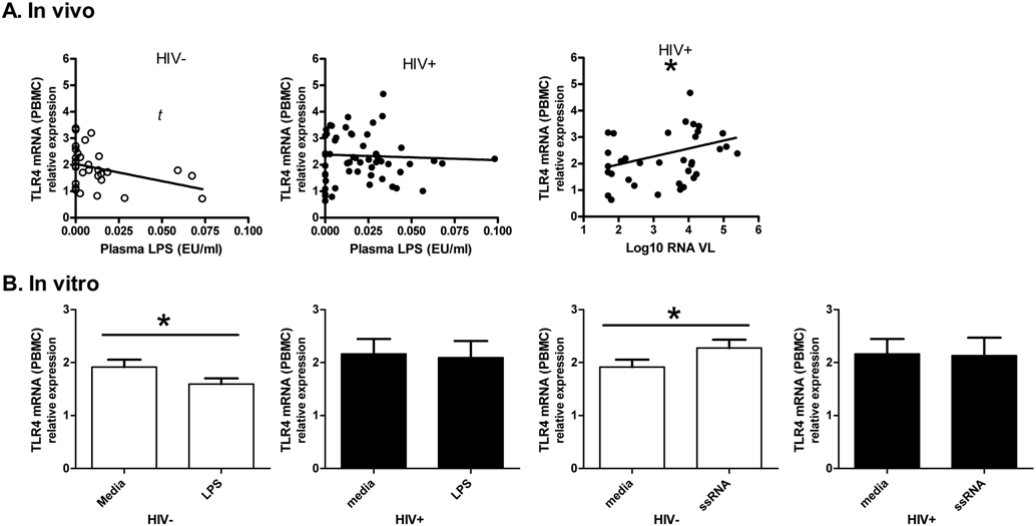
In vivo and in vitro associations between endotoxin (LPS) levels and TLR4 mRNA expression in PBMCs. (A) In samples obtained directly from HIV uninfected subjects, an inverse relationship between plasma LPS levels and TLR4 expression was apparent (R = −0.35, P = 0.053, Spearman Rank correlation). However, in HIV infected subjects no correlation or trend was seen (R = 0.029, P = 0.83). Additionally, plasma HIV RNA levels were positively associated with increased TLR4 mRNA levels (Spearman R = 0.3260, P = 0.049). (B) In a similar pattern, overnight culture with 0.1 ug/l LPS reduced TLR4 mRNA expression in PBMC of HIV uninfected (n = 70, P = 0.009), but not infected subjects (n = 21, P = 0.627). Isolated HIV ssRNA40 increased TLR4 mRNA expression in PBMC from HIV uninfected subjects (n = 70, P = 0.011), but not infected subjects (n = 21, P = 0.808). Spearman rank sum and Wilcoxon signed rank tests were used, two-tailed ns = not significant, *t* = trend(P<0.1),*P<0.05.

Endotoxin (LPS) sends proinflammatory signals primarily through TLR4. Since tolerance to endotoxin is important in preventing overwhelming inflammatory responses in advanced sepsis[15], decreased proinflammatory signaling through chronic or repetitive endotoxin exposure is expected. Here, we compared basal TLR4 mRNA expression levels with plasma LPS levels in matched plasmas and PBMCs from study subjects. In HIV uninfected subjects (Figure 2A), a negative correlation between TLR4 mRNA and plasma endotoxin levels was seen, however this did not reach statistical significance (R = −0.351, P = 0.053). However, in HIV infected subjects, no correlation or trend was observed (R = 0.0661, P = 0.694). As previously described, TLR4 mRNA from PBMC was significantly associated with increased HIV RNA load (Spearman R = 0.326, P = 0.049).

**Figure 3:**
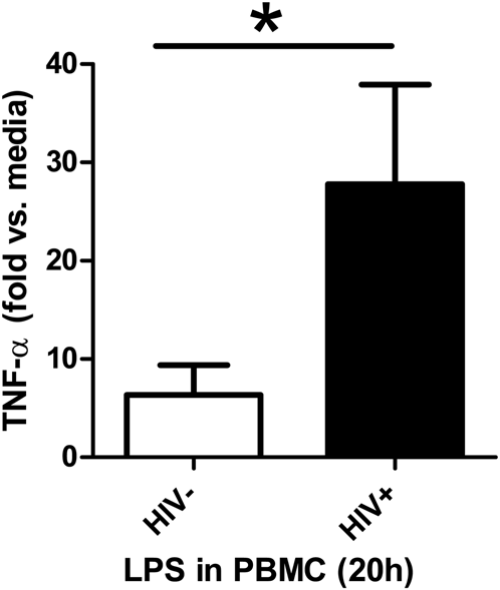
Effect of HIV-1 infection on proinflammatory cytokine responses to endotoxin (LPS) in PBMC. In PBMC from HIV uninfected (n = 10) and HIV infected (n = 9) subjects were cultured in media alone or in the presents of 0.1 ug/ml LPS overnight and TNF-α levels were determined in culture supernatants. Results are given as fold TNF-α increases in LPS vs. media stimulated cells. Comparison is by Mann-Whitney test. Two sided *P = 0.017.

In short term *in vitro* culture, similar patterns of changes in TLR expression were seen. Overnight LPS stimulation reduced TLR4 mRNA expression in PBMC from HIV uninfected subjects (Figure 2B, P = 0.009), but not from HIV infected subjects (P = 0.8078), who had a higher magnitude of TLR4 expression at baseline. To summarize this data, direct patient samples (*ex vivo*) and cell culture (*in vitro*) data suggest LPS functionally reduces TLR4 expression, but HIV ssRNA induces TLR4 expression in HIV uninfected subjects. However, this TLR4 expressional regulation is not observed in HIV infected subjects. As previously reported, (reshown in Figure 3 using different subjects), HIV infection was also associated with higher proinflammatory TNF-α responses in PBMC stimulated with LPS.

**Figure 4:**
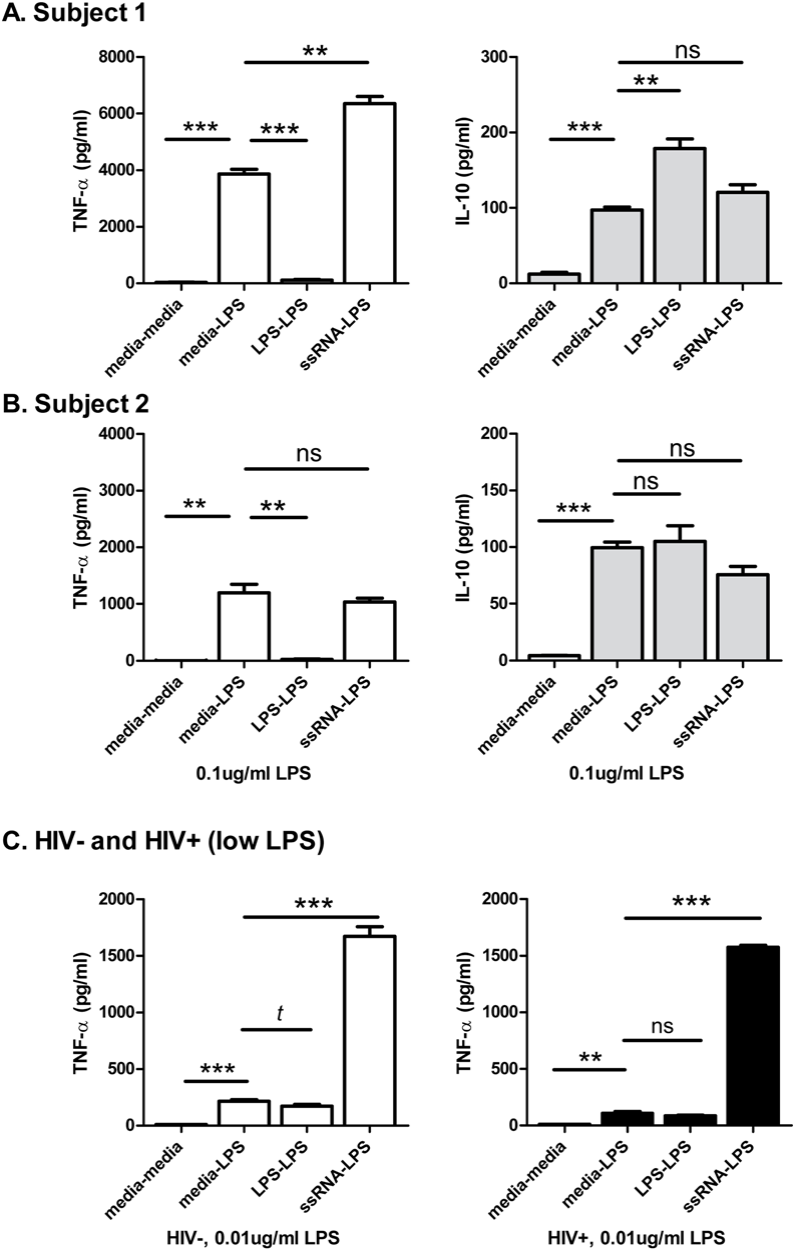
In vitro endotoxin (LPS) ‘tolerance’ and TLR ‘priming’ of cytokine responses by single-stranded RNA. Data for triplicate experiments in PBMCs from two HIV uninfected subjects are shown in (A and B) using 0.1 ug/ml of LPS, 1 ug/ml ssRNA40, or media alone for initial priming and subsequent stimulation steps (e.g. ‘media-LPS’). In the first subject, pre-incubating PBMCs with LPS dramatically reduced subsequent LPS-induced TNF-α responses (P<0.0001). However, pre-incubating with HIV ssRNA (RNA40) increased subsequent LPS-induced TNF-α responses (P = 0.0011) when compared with pre-incubating with culture media alone. Conversely, LPS pre-incubation resulted in higher endotoxin-induced IL-10 responses than either media or HIV ssRNA-primed endotoxin stimulations (P = 0.0039, P = 0.025, and P = 0.098). In a second subject (B), with lower overall cytokine production from PBMC, LPS priming also reduced subsequent LPS induced TNF-α production (P = 0.0015), but priming with ssRNA did not result in significantly altered TNF-α responses (P = 0.39). Also in the second subject, ssRNA primed PBMCs induced lower IL-10 to subsequent LPS stimulation than media primed PBMCs at borderline statistical significance (P = 0.056). (C) The same experiment design but with reduced LPS levels to 0.01 ug/ml include one uninfected and one HIV infected subject. Lowering the LPS dose to 0.01 ug/ml resulted a trend toward LPS tolerance in the uninfected subjects (P = 0.097), but no significant tolerance in the HIV infected subjects (P = 0.325). However, in both cases, pre-stimulating PBMC with ssRNA40 dramatically enhanced subsequent LPS induced TNF-α (P<0.0001 for both). Between priming and the subsequent LPS stimulations, PBMCs were washed to remove solublized cytokines; and carry-over cytokines tested were negligible. All experiments were run in triplicate. Unpaired two tailed t tests were used. Level of significance ns = not significant, *t* = trend(P<0.1), *P≤0.05, **P≤0.01, and ***P≤0.001.

To compare the ability of LPS versus HIV ssRNA to induce functional TLR4 ‘tolerance’ (reduced proinflammatory responses to LPS) or ‘priming’ (enhanced proinflammatory responses to LPS), we performed a series of experiments as depicted in Figure 4. Considerable variability in cytokine response levels was seen between subjects and with various doses of LPS, but intra-subject responses were consistent. Two patterns of responses, using the same LPS dose as the TLR expression experiments above, 0.1 ug/ml LPS, were observed as shown in Figure 4A and 4B which depicts two experiments on PBMC from HIV uninfected subjects, each run in triplicate. Shown first, pre-incubating with LPS dramatically reduced subsequent LPS-induced TNF-α responses (3% of media-primed response, P<0.0001). On the other hand, pre-incubating with HIV ssRNA significantly enhanced LPS-induced TNF-α responses (164% of media-primed response, P = 0.0011). In contrast to the TNF-α findings, production of the immunoregulatory cytokine IL-10 was higher when pre-incubated with LPS (184% of media-primed response, P = 0.0039). Pre-incubating with HIV ssRNA and stimulating with LPS led to a statistically non-significant trend toward increased IL-10 production compared to media-primed responses (P = 0.098). The second subject shown (Figure 4B) demonstrated similar LPS-induced tolerance (P = 0.0015), however, ssRNA primed TNF-α responses were not significantly different from media-primed responses (P = 0.385). In this subject, HIV ssRNA priming trended toward reducing the magnitude of the IL-10 response to LPS (P = 0.056). To assess the effect of endotoxin tolerance and reduced LPS levels, we reduced the dose of LPS for stimulations to 0.01 ug/ml (Figure 4C). In this case, LPS induced TLR tolerance was only a trend in an HIV uninfected subject, and not significant in an HIV infected subject. However, in both cases, pre-incubating with ssRNA resulted in greatly enhanced LPS-induced TNF-α responses (P<0.001 for both). Therefore, *in vitro*, LPS induced proinflammatory tolerance to repeat LPS stimulation, at least at higher doses, but HIV ssRNA often increased proinflammatory responsiveness to LPS in PBMC (TLR priming).

## Discussion

The data in these studies support that innate immune responses are fundamentally altered in HIV infection, which may influence chronic immune activation status and HIV disease. We previously identified marked changes in TLR levels and signaling associated with HIV infection[13]. Here we focused on the effect of HIV infection and HIV RNA on endotoxin signaling. ‘Microbial translocation’ of bacterial products from a disturbed gastrointestinal tract has been proposed as a driver of HIV-associated chronic immune activation[2], which in turn predicts progression to AIDS[16]. Here, we confirmed, for the first time in an African setting, the association between chronic HIV infection and elevated plasma endotoxin levels. Interestingly, clinical symptoms or conditions that could indicate acute underlying infections where more frequent in HIV infected subjects, but asymptomatic HIV infected subjects were at least as likely to have high plasma endotoxin levels as those with symptoms or a diagnosed acute infection. This is supportive of a chronic, sub-clinical cause for ‘walking endotoxemia’ in HIV infected individuals[16].

It is difficult from our study to determine the origin of plasma endotoxin in non-HIV infected subjects. On further review of the three HIV-uninfected subjects with the highest endotoxin levels, one had a skin abscess (of which the causative agents are usually Gram positive, thus not LPS producing), one had bacterial vaginosis (which is non-invasive and unlikely to result in bacteremia), and the other had no identifiable illness at presentation. No other acute infectious etiologies were associated with detectable plasma endotoxin levels. Other underlying endemic infections such as with intestinal helminthes could be present and easily missed[17], however. Additionally, vaginal douching with detergents is a common practice of sex-workers in Kenya[18], which could hypothetically disrupt the genital mucosa and affect microbial translocation at this site, though this has not been previously shown. Further studies are required to assess the causes of subclinical endotoxemia in this and other populations, and the consistency of these findings over time.

We assessed the effect of HIV infection and an HIV RNA analogue on the central immune signaling receptor for LPS, TLR4. Immune tolerance to overwhelming inflammatory stimuli, as seen in overt sepsis[19], is necessary to prevent circulatory collapse and death. In the case of chronic endotoxemia, little is known about immune control mechanisms. Here we show, for the first time, that chronic TLR4-mediated endotoxin tolerance is likely, since (at the cusps of statistical significance) plasma endotoxin levels were associated with decreased TLR4 mRNA levels in peripheral blood, at least in HIV uninfected subjects. This was further supported by *in vitro* data whereby PBMC culture in the presence of LPS, significantly lowered expression of TLR4 mRNA compared to media controls. Conversely, as we previously demonstrated, plasma HIV RNA levels in HIV infected subjects were associated with increased TLR4 mRNA levels in PBMC. This TLR4 mRNA enhancement was supported by *in vitro* culture data using short HIV- single stranded RNA analog (ssRNA40). Therefore, since both HIV viremia and low level endotoxemia are likely to be persistently present in blood of HIV infected subjects, with each appearing to have an opposing effect on TLR4 levels, one could expect an altered TLR response in HIV infection. Indeed, TLR4 mRNA levels in PBMC of HIV infected subjects started at higher basal levels, and did not show evidence of expressional tolerance on exposure to additional LPS or ssRNA.

A greater proinflammatory (TNF-α) response to LPS was associated with HIV infection in this study, as previously reported[13]. To further evaluate the effect of HIV RNA on endotoxin signaling, and TLR4, we pre-stimulated PBMC with HIV ssRNA or LPS, before additional LPS stimulation. As expected, pre-stimulation with LPS resulted in decreased TNF-α production to subsequent LPS, compared to pre-stimulation with media alone, indicating tolerance. On the other hand, pre-stimulating PBMC with HIV ssRNA in most subjects resulted in higher TNF-α production to subsequent LPS compared to media pre-stimulated controls. This provided further evidence that HIV RNA was independently able to enhance the proinflammatory response to endotoxin. Interestingly, others have shown that LPS can drive HIV replication, even in the face of tolerized endotoxin responses[20].

The mechanism by which HIV RNA influences TLR signaling requires further evaluation. Feedback TLR expression, as described here is one mechanism of immune regulation previously described[21], but other mechanisms exist[22], [23]. Also, production of immune regulatory cytokine, IL-10, was induced at higher levels in response to LPS than RNA40 in some subjects, but this was not observed in others. Relative proportions of cell subsets may also contribute to relative TLR quantities and responsiveness. Although PBMC contain a mixture of immune cell types, each possessing a variable expression of TLR, the overall responses to endotoxin and HIV RNA appeared consistent and functionally relevant. Further studies are warranted to delineate the TLR signaling interactions in cellular subpopulations and tissue dependence of cross-TLR signaling.

The implication of altered TLR responsiveness to endotoxin in HIV infection is substantive. Chronic immune activation is a strong predictor of HIV disease and death. Meier et al. suggested that HIV RNA may lead to immune activation directly through innate immune recognition by TLR8[10], and they demonstrated that treatment with ART rapidly reduced immune activation levels, paralleling HIV RNA suppression[12]. On the other hand, Brenchley and others suggested that microbial translocation and plasma endotoxemia are a cause of immune activation in HIV[24]. In this latter case, the source of inflammatory ligand is an indirect effect of HIV infection, and ART suppressive therapy appears to only gradually and partially lead to correction of the underlying process[25], [26]. At this time that process is thought to be gastrointestinal mucosal integrity. Importantly, if microbial stimuli such as plasma LPS are drivers of immune activation and ultimately contribute to CD4 T cell decline and HIV disease, then viral suppressive therapy would only be expected to have limited or delayed affect on CD4 recovery and prevention of opportunistic infections. While this is sometimes the case, our data suggest that if HIV RNA is a cause of enhanced proinflammatory response to endotoxin, then by removing circulating HIV RNA, such as with ART, normal tolerance to LPS could potentially be restored. In other words, the response to persistent endotoxemia could be dampened to normal or near normal levels. Indeed, many subjects respond with rapid immune reconstitution to ART, in a time frame unlikely to result from gastrointestinal mucosal repair, and may result in part from reduced innate immune dysregulation caused by circulating HIV RNA.

New techniques have enabled the detection of minute quantities of diverse bacterial products in plasma and other typically sterile sites[26], [27]. The precise mechanisms, extent and effects of microbial translocation in HIV are only starting to be worked out[25], [28], [29], [30], [31]. Similarly, innate immune changes in acute and chronic HIV infection are much less understood than their adaptive immune counterparts[32]. This study indicates that innate immune signaling, through TLR, is altered in HIV infection. Specifically, HIV RNA may influence the response to microbial translocation through TLR4 signaling, and promote or enable the cycle of immune activation and burn out that ultimately contributes to HIV immune dysfunction and AIDS. Better understanding these mechanisms may lead to new approaches to HIV and AIDS control.
